# Out-of-sync: disrupted neural activity in emotional circuitry during film viewing in melancholic depression

**DOI:** 10.1038/srep11605

**Published:** 2015-06-26

**Authors:** Christine C. Guo, Vinh T. Nguyen, Matthew P. Hyett, Gordon B. Parker, Michael J. Breakspear

**Affiliations:** 1 QIMR Berghofer Medical Research Institute, Herston, Queensland, Australia; 2 School of Psychiatry, University of New South Wales, Sydney, Australia; 3 Black Dog Institute, Sydney, Australia; 4 Metro North Mental Health Service, Herston, QLD,4009,Australia

## Abstract

While a rich body of research in controlled experiments has established changes in the neural circuitry of emotion in major depressive disorders, little is known as to how such alterations might translate into complex, naturalistic settings - namely involving dynamic multimodal stimuli with rich contexts, such as those provided by films. Neuroimaging paradigms employing dynamic natural stimuli alleviate the anxiety often associated with complex tasks and eschew the need for laboratory-style abstractions, hence providing an ecologically valid means of elucidating neural underpinnings of neuropsychiatric disorders. To probe the neurobiological signature of refined depression subtypes, we acquired functional neuroimaging data in patients with the melancholic subtype of major depressive disorder during free viewing of emotionally salient films. We found a marked disengagement of ventromedial prefrontal cortex during natural viewing of a film with negative emotional valence in patients with melancholia. This effect significantly correlated with depression severity. Such changes occurred on the background of diminished consistency of neural activity in visual and auditory sensory networks, as well as higher-order networks involved in emotion and attention, including bilateral intraparietal sulcus and right anterior insula. These findings may reflect a failure to re-allocate resources and diminished reactivity to external emotional stimuli in melancholia.

Deficits in emotion and cognition in major depressive disorder (MDD) disrupt interpersonal functioning, causing difficulties across a wide range of everyday tasks^1^. Patients with melancholia, a refined subtype of MDD with prominent neurobiological underpinnings, have particularly pronounced deficits in emotional cognition and marked impairments in social and interpersonal functioning^2^,^3^. Non-invasive neuroimaging technologies permit *in vivo* characterization of the anatomical, physiological and neurochemical correlates of MDD^4^ and hence provide insight into the correlates of social and emotional dysfunction. Using carefully designed tasks, such research has established that MDD is associated with disturbances in ventromedial prefrontal cortex (vmPFC), anterior cingulate cortex (ACC), insula cortex and closely related subcortical structures^4^,^5^. Importantly, the activation and functional connectivity of subgenual ACC and vmPFC have been shown to correlate with depression severity^6^,^7^,^8^,^9^,^10^,^11^. A rich body of neuroimaging studies in healthy participants has established that these brain structures represent key neural circuits in processing and modulating emotional experience (sFig. 1, sTable 1). For example, the anterior insula has been widely implicated in the feeling of disgust and the subgenual ACC in the induction of sadness^12^,^13^. Therefore, it seems reasonable to extrapolate these alterations in emotion-modulating circuits occurring in controlled tasks to disturbances in mood and function experienced by people with MDD in everyday life. However, this link remains conjectural, pending examination of neural responses during natural, realistic emotional experiences.

The study of cognition and perception during realistic, natural conditions has attracted increasing attention in neuroimaging, benefitting from rapid developments in analytical methodology^14^. Using inter-subject correlation (ISC) analysis, a series of innovative neuroimaging studies have recently shown that naturalistic stimuli, such as free viewing of films, evoke highly consistent responses in many cortical regions across subjects, despite the seemingly uncontrolled nature of such paradigms^15^,^16^,^17^,^18^,^19^,^20^,^21^,^22^. Visual areas known to be specialized for object or face perception appear to be reliably activated when objects or faces appear within film streams^15^,^22^. Building on these early studies, natural stimuli such as films, music and narratives are being increasingly employed in neuroimaging experiments, providing novel insights into the neural processes underlying perception and language in more natural contexts^20^,^23^,^24^,^25^,^26^. Inter-subject consistency otherwise tends to be relatively weak in higher-order brain regions, posing a challenge to the study of abstract processes. Such a barrier might be overcome by selecting natural stimuli that specifically engage higher-order processes, such as well-directed films which are carefully constructed in order to elicit and manipulate our feelings and emotions. In a recent study, an Academy Award-winning emotionally salient film has been shown to induce robustly consistent neural responses in emotional circuitry including the anterior cingulate cortex and anterior insula^18^.

Functional neuroimaging acquired during film viewing offers an ideal experimental paradigm to study neural circuits during realistic emotional experiences, and their disruption in mental illness. As minimum training or in-scanner performance is required, this approach enjoys similar advantages to resting state acquisitions and thus holds great potential for imaging patient populations, minimizing anxiety associated with completing difficult or repetitive tasks. On the other hand, natural stimuli put ecologically relevant constraints on neuronal processes and are thus more likely to selectively engage brain networks of interest than resting state acquisitions. Here, we acquired fMRI data while patients with the melancholic subtype of MDD (henceforth melancholic-MDD) and age-matched healthy controls freely viewed film clips with different emotional salience (Table 1). We chose to study patients with melancholia as it has long been positioned as reflecting the quintessential depressive disease, possessing strong biological underpinnings^2^,^3^. Those with melancholia also represent a more homogeneous clinical group in comparison to those with (non-melancholic) MDD, which is more a domain diagnosis capturing multiple and heterogeneous conditions with quite variable psychological, social and biological determinants. We hypothesized that melancholic patients would exhibit diminished consistency in brain dynamics, specifically to emotional films, and that these effects would be strongest within the neural circuits of emotion. We analyzed our fMRI data using both voxel-wise inter-subject correlations (ISC), as well as using multivariate techniques to study the consistency of large-scale networks.

## Results

We analyzed fMRI data from 23 healthy and 17 participants with melancholia who viewed two emotionally salient film clips and one neutral film. The positive emotional film was an out-take from a stand-up comedy show by Bill Cosby, a well-validated stimulus shown to consistently elicit smiling and laughter^27^. The negative emotional film was a dramatic and disturbing segment from “The Power of One”, depicting slaves being degraded by soldiers. We first validated the specific emotional valence (positive and negative, respectively) of each of these films in a second group of healthy participants who rated these films dynamically during an independent viewing (sFig. 2; see Methods for details). The overall assessment of these films was highly consistent between participants, as was the dynamic, time dependent rating. The neutral film showed scenes from an airplane flying over natural landscapes (mountains) and shots of rivers with an airplane engine and running water sounds, chosen to minimize emotional responses.

## Voxel-wise ISC in emotion-processing regions are reduced in MDD

**Figure 1:**
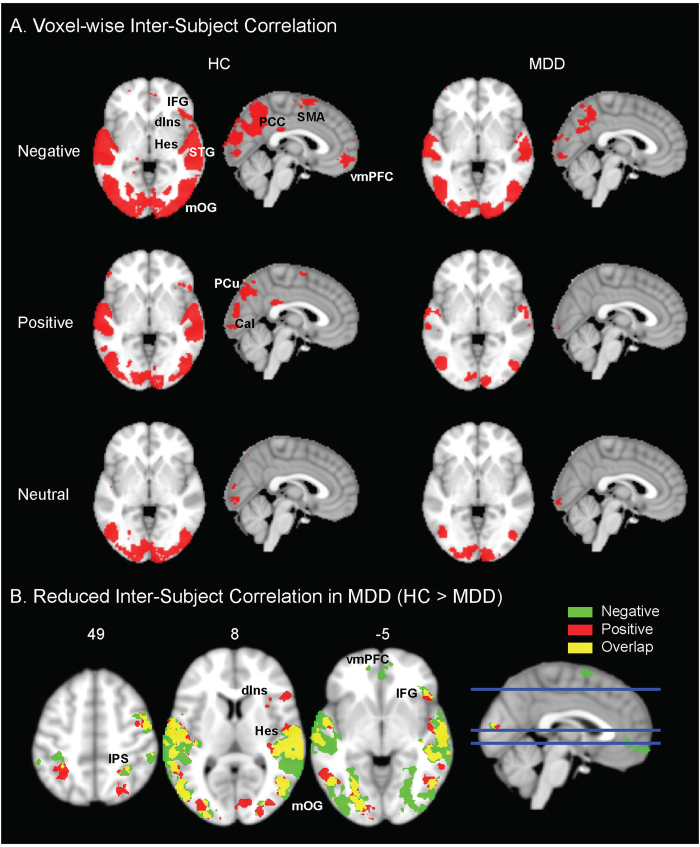
Voxelwise ISC maps in HC and MDD. (**A**) Group-level ISC maps for HC (left) and MDD (right) during natural viewing of negative, positive and neutral films (p < 0.01, FDR corrected). (**B**) Group difference maps (MDD < HC) of ISC results for the negative (green) and positive (red) films, and the overlap between the two viewing conditions (yellow; p < 0.05, FDR corrected; cluster size > 50 voxels). Calc = calcarine; dIns = dorsal insula; IFG = inferior frontal gyrus; IPS = intraparietal sulcus; Hes = Heschl’s; mOG = middle occipital gyrus; PCC = posterior cingulate cortex; PCu = precuneus; SMA = supplementary motor area; STG = superior temporal gyrus; vmPFC = ventromedial prefrontal cortex.

Free viewing of these films evoked robust and consistent neural activity, as quantified by the inter-subject correlation (ISC) of voxel-wise BOLD signals (Fig 1a, left). Robust inter-subject synchronization in our healthy participants was evident in primary and association visual areas during all three film-viewing conditions. However, consistent responses across more widespread regions were also elicited by the two emotionally salient films, encompassing primary and association auditory cortices, fusiform gyrus (FUS), parahippocampal gyrus (PPA), precuneus (PreC), and supplemental motor area (SMA). These responses extended into higher-order brain regions, including posterior cingulate cortex (PCC), bilateral intraparietal sulcus (IPS), right anterior dorsal insula (dIns) and inferior frontal gyrus (IFG). Notably, the negative film consistently engaged a cluster in the ventromedial prefrontal cortex (vmPFC) in the healthy subjects (Fig. 1a, upper left).

The consistency of neural responses was substantially diminished in the data from our patients with melancholic-MDD during free viewing of emotionally salient films (Fig. 1b, sFig. 3; *p* < 0.05, FDR corrected). This reduction encompassed sensory association areas, including medial occipital gyrus (mOG) and superior temporal gyrus (STG), as well as high-order brain regions, including bilateral IPS, right anterior dIns and IFG. Intriguingly, the responses of vmPFC were significantly reduced in patients with melancholic-MDD specifically during free-viewing of the negative film (Fig. 1b, sFig. 3). Following recent statistical guidelines, we also calculated the Bayes Factor of these results (see Supp. Inf). This analysis yielded a Bayes Factor of 11.7, which corresponds to strong evidence for the alternative hypothesis - namely of a robust difference in the voxel-wise ISC maps between HC and MDD participants. In contrast, there were no significant reductions in ISC during viewing of the neutral film.

## Tensor ICA reveals large-scale functional networks during free film viewing

We next used a multivariate approach, tensor independent component analysis (tICA), to extract large-scale network patterns that were engaged during natural viewing. Within each film condition, tICA identifies distributed brain regions whose neural activities co-vary across participants, without *a priori* assumptions of the stimulus profile^28^. Hence, tICA is particularly suitable for extracting neural networks that are consistently engaged during natural viewing conditions. After excluding noise components primarily encompassing non-gray matter voxels, roughly half of the tICA components in our data were identified as candidate functional networks, based on their spatial patterns, with the highest percentage during negative film viewing (46%) and the lowest percentage during neutral film viewing (34%).

**Figure 2:**
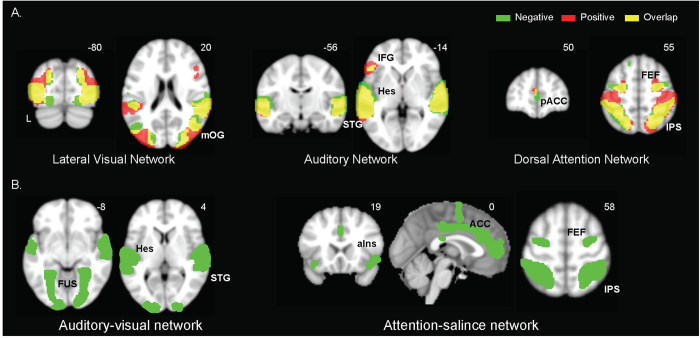
Selected network components from tensor ICA analysis. (**A**) Network components present during viewing conditions of the negative (green) and positive (red) films, and the overlap (yellow). (**B**) Network components present during viewing of the negative film. ACC = anterior cingulate cortex; aIns = anterior insula; FEF = frontal eye field; FUS = fusiform gyrus; IFG = inferior frontal gyrus; IPS = intraparietal sulcus; Hes = Heschl’s; mOG = middle occipital gyrus; pACC = posterior anterior cingulate cortex; PCC = posterior cingulate cortex; PCu = precuneus; SMA = supplementary motor area; STG = superior temporal gyrus.

Several of these functional network components showed similar patterns to those identified in resting state fMRI data, confirmed through spatial cross-correlation with previously published templates and visual inspection^29^,^30^,^31^. This similarity in network patterns possibly reflects intrinsic neural processes and connectivity during both film-viewing and resting state conditions (Fig. 2a; neutral film condition not shown)^32^. While most of these networks were present across all three viewing conditions, the dorsal attention network was not present during neutral film viewing, possibly because the neutral film failed to consistently capture the attention of our participants. Some of the networks appear to be unique to natural viewing conditions, and showed intriguing functional selectivity to scenes during the film streams (Fig. 2b). In the next section, functional selectivity was examined for three network components extracted from the negative emotional film.

## Functional selectivity of large-scale neural networks during natural viewing

**Figure 3:**
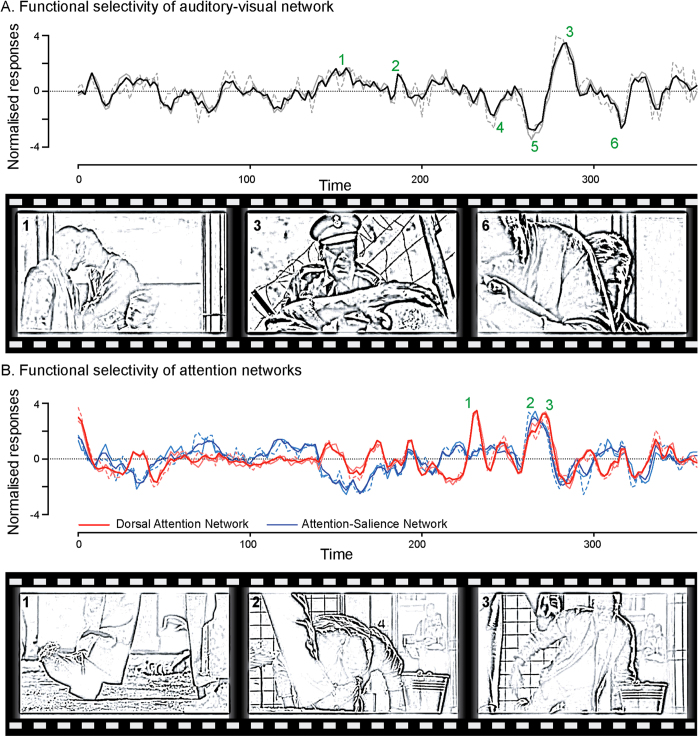
(**A**) Functional selectivity of auditory-visual network. (**B**) Functional selectivity of dorsal-attention network (red) and attention-salience network (blue). Upper panel shows the time course of auditory-visual network during viewing of the negative film, averaged across all participants (thick line), healthy controls (thin line) and patients with MDD (thin dashed line). Scenes corresponding to certain points of the time course are signified by green numbers and shown in the lower panel. region. [Movie stills courtesy of Warner Home Video. The Power of One © 1992 Regency Enterprises V. O. F. & Le Studio Canal + All Rights Reserved.]

The temporal profile of functional network activity revealed that distributed cortical regions were collectively engaged during selective aspects of complex natural scenes. One representative network of note is an auditory-visual network (component 10) derived from the negative film viewing condition. This network encompasses both auditory regions, including STG and Heschl’s gyri, and the ventral visual stream, including FUS (Fig. 2b). Based on these anatomical substrates, we hypothesized that this network might be consistently engaged during conversational scenes when both human faces and voices were present. Consistent with our hypothesis, the three highest peaks of its time course occurred during conversation (Fig. 3a, scene no. 1, 2, 3), whereas the three troughs of its time course occurred during periods of silence, even when faces were present (Fig. 3a, scene no. 4, 5, 6). Formal statistical analysis revealed that the temporal profiles of the audio-visual ICA components were robustly correlated with the co-occurrences of human face and voice in the film streams (sFig. 4). Furthermore, this correlation was significantly higher among healthy participants than patients with melancholic-MDD (sFig. 4).

Observing the activity of this sensory network motivated us to examine networks encompassing higher-order cortical regions. Component 5 from the negative film viewing condition had a spatial distribution consistent with the dorsal attention network (Fig. 2a)^33^. Its time course shows a peak near the beginning of the film, suggesting participants consistently directed their attention to the film upon its commencement. The time course of the network peaks again later, as the film evolves toward the most emotionally charged scenes, depicting scenes of the soldier degrading the prisoner (Fig. 3b, scene no. 1 – soldier shows his shoe and no. 2 – soldier kicks the prisoner away). Interestingly, a similar ICA component (component 7), encompassed the salience network in addition to the dorsal attention network^29^, with the peak occurring at the most emotionally disturbing scene (no. 3) as rated by independent viewers (sFig. 2). Such a response is consistent with the function of the salience network, particularly the anterior insula, in signaling strong emotional salience (here disgust)^12^,^34^,^35^.

## ISC of functional network dynamics are reduced in melancholia

**Figure 4:**
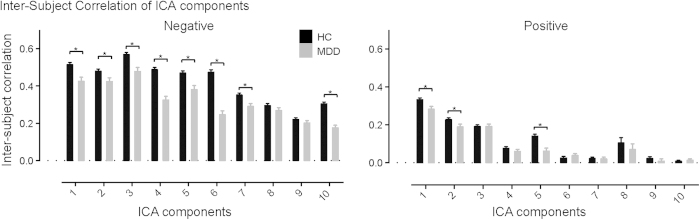
Group average of inter-subject correlation of ICA component time courses for HC (black) and MDD (gray) during viewing of the negative (left) and positive (right) films. Asterisks signify significant differences between HC and MDD (p < 0.05, FDR corrected).

The dynamics of functional networks derived with tICA exhibited diminished inter-subject consistency in melancholic-MDD participants during the emotional films. In particular, we tested for group differences in ISC for the first 10 network components from each film viewing condition, controlling for multiple comparisons using false discovery rate (FDR). ISC was significantly reduced in melancholic-MDD for 8 out of the 10 network components during negative film viewing, and 3 network components during positive film viewing (Fig. 4; *p* < 0.05, FDR corrected). There were no significant reductions during neutral film viewing. This pattern of group differences (strong effects for the negative film, and negligible for the neutral film) converged with the results from voxel-wise ISC analyses, suggesting that melancholia is associated with substantial disturbances in the neuronal underpinnings of negative emotional experience.

## Abnormal vmPFC activity correlates with depression severity

**Figure 5:**
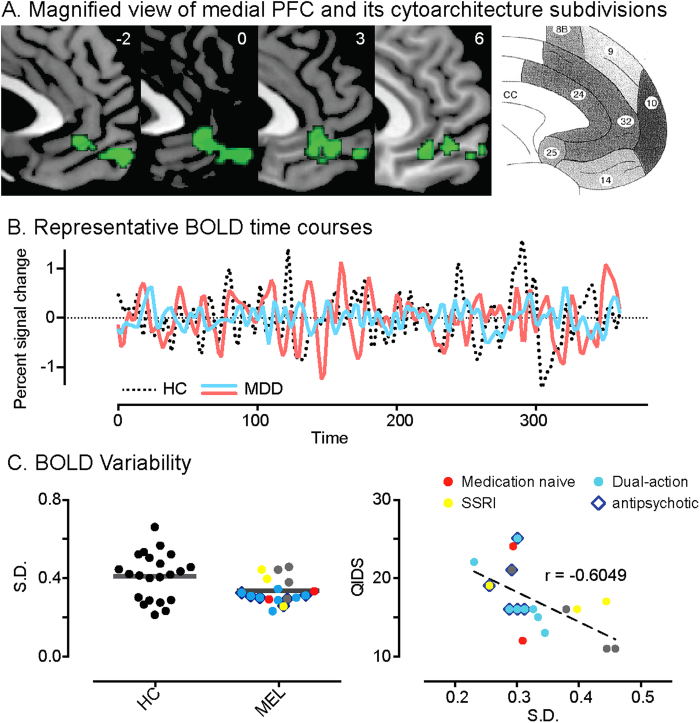
(**A**) Magnified view of the medial PFC highlighting the cluster that shows a significant difference in ISC between HC and MDD (Fig. 1B), and its cytoarchitecture subdivisions (far right; reprint from (35)). (**B**) Representative traces of BOLD time course from this cluster from one HC (dashed) and two patients with MDD (blue and red). (**C**) Standard deviation of BOLD time courses between HC and MDD (black bars signify the mean; left panel); Standard deviation plotted against QIDS in MDD (right panel). The example participants in B are colour coded to illustrate their distribution in the group.

Previous neuroimaging and lesion studies have suggested task-related functional impairments of vmPFC (Brodman areas 10, 25 and 32) in MDD^11^,^36^. As described above, our voxel-wise ISC analysis revealed a diminished inter-subject consistency in vmPFC during negative film viewing in the depressed melancholic-MDD participants (Figs 1b and 5a, also see sFig. 3). We next explored whether the activity of this cluster co-varied with the clinical severity of the depressive episodes. Visual inspection of the BOLD time series of this vmPFC cluster suggests more dynamic responses in HC than melancholic-MDD (Fig. 5b). Quantifying the variability of these dynamics by their standard deviation indeed showed a significantly stronger variability in the HCs than those with melancholia (Fig. 5c; two-sample test, *p* = 0.02). Crucially, within our depressed group, this variability was negatively correlated with QIDS scores, a standardized clinical instrument measuring depression severity (r = −0.60; *p* = 0.005). This correlation remains significant when age, gender, current smoking status, age at onset, duration of current episode and family history are included as covariates (r = −0.65; *p* = 0.015). We note that this (correlation) analysis is independent of the original whole brain search (which did not incorporate symptom severity in any way). BOLD signal variability in voxels outside of the ISC difference map did not differ significantly between melancholic-MDD and HC participants (*p* > 0.05), and were not correlated with depression severity (r = −0.18; *p* > 0.2). Such specificity suggests that the differences in neural activity were not due to physiological confounds or motion artifacts. Visualizing the distribution of the data points from patients with their various medications, shows a lack of clustering by medication class, suggesting that these results were unlikely to have been driven by specific medication effects (Fig. 5c).

## Discussion

By capturing the specific deficits in emotional circuit activity during free viewing of emotionally salient film clips, our findings bring fresh insights to the functional neurobiology of the melancholic sub-type of major depressive disorder. Our work recasts established findings using controlled stimuli into a more ecologically valid context. We observed a range of context-specific changes in sensory cortices, including FUS, PPA, mOG and STG, as well as higher order brain regions such as vmPFC, anterior insula and IPS – key regions in emotion processing and salience detection^35^,^37^. We also observed a striking correlation between the reduction in vmPFC dynamics during negative film viewing and depression severity in our melancholic participants. These findings are in accordance with previous reports that subgenual ACC and vmPFC show lower gray matter volume and cell counts, as well as abnormal glucose metabolism and cerebral blood flow in major depression^4^, and that these regions represent key neural substrates in emotion-modulating networks, and arguably encode sadness^12^,^13^,^38^.

Naturalistic paradigms, such as films, offer enormous potential to reveal neural processes underlying dynamic emotional responses and their perturbation in neuropathological conditions. Such designs could be more effective in revealing brain dysfunction than resting state paradigms, which have been used extensively over the last ten years to study neuropsychiatric disorders^39^. Several resting state fMRI studies have addressed the integrity of ACC and vmPFC in depressed subjects. These findings, however, remain inconclusive regarding the neural origins of emotional disturbances^5^,^7^,^40^,^41^,^42^,^43^,^44^. While most resting state studies have found changes in ACC and vmPFC, some have reported increased functional connectivity of these regions^5^,^40^,^41^,^43^,^44^, whereas others reported decreased connectivity^7^,^42^. These conflicting results could be due to the unconstrained nature of resting state paradigms, the nuances of the specific “no task” instructions, patient selection or other confounds. Functional neuroimaging research that deploys free viewing still benefits from the minimum demand for training and task execution as do resting state paradigms, but confers better control over the timing, recruitment and coordination of neural processes. We here established its value as an effective and ecologically valid tool to examine brain dysfunction in melancholia, a canonical neuropsychiatric disorder. By specifically selecting films with high emotional salience, we found that emotion-modulating regions, such as vmPFC and right AI, are consistently engaged in healthy subjects and impaired in those with melancholic-MDD, with a particular failure to engage key regions during the emotional peaks of the film. These findings were not evident during the neutral film condition.

The deployment of both voxel-wise and multivariate analyses using tensor ICA revealed convergent findings. Tensor ICA provides a novel method to extract large-scale functional networks driven by natural stimuli. In contrast to the use of spatial ICA in previous neuroimaging studies with natural stimuli^18^,^21^, tICA is designed to identify network components with common underlying temporal profiles between individuals^28^. Accordingly, the ranking of these components is based on the robustness of their engagement across subjects. Network components with high tICA ranking tend to show high inter-subject consistency, likely reflecting neural processes that were specifically engaged by the film streams. Furthermore, this engagement seems to be functionally selective for distinctive aspects of complex natural experience (Fig. 3, sFig. 4). On the other hand, some network components with low tICA ranking share a similar spatial distribution to networks identified at rest, confirming the intrinsic nature of these connectivity networks, which are preserved under natural viewing conditions. These findings highlight the elusive nature of functional brain networks - their support of internally generated cognitive processes, but also their execution of perceptual processes during natural experience – an intriguing area for further research.

The reduced consistency in neural responses to the negative film in melancholic-MDD could reflect the altered internal processing of negative emotion – patients with depressive disorders, although they share a common diagnosis, are likely to process negative stimuli in distinctive ways that are unique to their personal experience, resulting in reduced consistency in neural responses to negative stimuli. Further, the robust correlation between the reduction in vmPFC dynamics during film viewing and the severity of the depressive episode suggests that these regions are stuck – or *dwell* – in non-adaptive states in melancholic individuals, and are unable to engage dynamically as they do in healthy control participants. The reduced variability in this key region also mirrors the subjective reports of those with this classic sub-set depressive condition, namely an impoverished cognitive experience, with persistent dysphoria that is unable to be shifted by external stimuli, either positive or negative in content – namely anhedonia. It is possible that the disturbances in perceptual regions in our results might be secondary, subject to impaired top-down control from vmPFC. Imaging analysis using effective connectivity could shed further insights on these possibilities.

Patients with melancholia were on antidepressant and/or mood stabilizer and antipsychotic medications. It is thus possible that our findings are confounded by medication. Clinical research on melancholic depression is challenged by a number of barriers to control for this – the severity of the disorder prohibits withholding of medication for research purposes, whilst the variety of classes and pharmacological targets prevents the possibility to estimate a class-invariant dosage equivalent. We have visualized the distribution of data points from patients using various medications (Fig. 5c). The lack of clustering by medication class argues against specific medication influences underlying our main group effects. The presence of a variety of medications from different classes also argues against an effect driven by the action of any single pharmaceutical agent.

Physiological responses are an integral part of emotional experiences^45^. Patients with major depression and other psychiatric conditions show altered physiological activity and reactivity^46^,^47^. Future imaging studies that incorporate simultaneous physiological recordings with the use of dynamic naturalistic stimuli would deepen our understanding of the physiological correlates of our findings. Another caveat of our study is that emotion valence is confounded by other features embedded in film stimuli. The three films were designed to evoke distinct emotional experiences of a positive, negative and neutral nature. However, other factors, in addition to valence, likely contributed to our results. For example, the negative clip we used is a segment taken from a major film, whereas the positive clip is a segment from a one man stand-up comedy performance: Both differ substantially in many low level features such as lighting, colour contrast, physical movement and sound. To address these potential confounds and pinpoint the specific effects of emotion, multiple film stimuli are needed to balance valence with these other film features. Using very short film clips might facilitate this goal^48^. However, we chose film clips of sufficient length to enable recognition of the underlying narrative and engage higher-order brain regions^19^,^26^. Due to these caveats, we did not focus on direct contrasts between the film conditions. We do discuss the intriguing observed differences evoked by positive and negative films, in the hope that these analyses inspire further studies to formally address the interaction between emotional valence and depression phenotype.

In sum, using naturalistic, emotionally salient material, we highlight several key regions whose activities showed diminished consistency and variance in patients with melancholia. Such results - and the simple approach of showing movies to patients - has considerable clinical potential; assisting diagnosis, monitoring response to treatment and, possibly, even planning brain stimulation approaches in patients with treatment-resistant illness.

## Methods and Materials

### Ethics statement

The study was carried out in accordance with the approved protocol by the University of New South Wales ethics committee (HREC 08077), and all subjects provided written informed consent prior to participation.

### Participants

Functional neuroimaging data were acquired from forty participants (17 patients and 23 healthy controls) for this study. Seventeen patients (9 females; aged 26–63) with major depressive disorder (MDD) were recruited through the specialist depression clinic at the Black Dog Institute in Sydney, Australia. Twenty-three right-handed age matched healthy controls (HC; 12 females; aged 22–75) were recruited from the Sydney community, and who disavowed lifetime or current mood and/or psychotic illness. All participants were screened for mood and psychotic conditions with the Mini International Neuropsychiatric Interview (MINI). Clinical MDD participants comprised those meeting criteria for a current major depressive episode, but without lifetime (hypo)mania or psychosis being identified during the MINI interview. We restricted our analyses to patients diagnosed with melancholic depression, made by clinic psychiatrists using previously detailed criteria^2^,^3^. In brief, patients with a diagnosis of melancholia were weighted toward the presence of a psychomotor disturbance (impaired concentration, motor slowing and/or agitation, anergia), an anhedonic and non-reactive mood, and with mood and energy showing a diurnal variation – being worse in the morning. Illness correlates including prior response to antidepressant medication and/or electroconvulsive therapy (ECT), a family history of depression or bipolar disorder, and episodes being more severe and persistent than any antecedent stressors, also weighted assignment of a melancholic diagnosis. Most patients were in receipt of multiple antidepressants, mood stabilisers and/or antipsychotics (Table 1). Formal diagnostic criteria are given in Supplemental Information.

Exclusion criteria for all participants were a current or past drug or alcohol dependence, ECT in the past six months, neurological disorder (i.e. neurodegenerative conditions, stroke, CNS infection, tremor), brain injury (i.e. neurotrauma from haemorrhage, hypoxia), invasive neurosurgery, and/or an estimated full scale IQ (WAIS-III) score below 80 as assessed by the Wechsler Test of Adult Reading (WTAR).

### Clinical Assessments

Depression severity in the clinical group was quantified with the Quick Inventory of Depressive Symptomatology or QIDS-SR^49^. Participants completed the State-Trait Anxiety Inventory (STAI), and overall functioning was measured by the Global Assessment of Functioning scale^50^. Psychomotor disturbance in the depressed participants was assessed by the CORE, a measure which provides a quantitative index of the level and severity of psychomotor disturbances - with non-interactiveness, retardation and agitation sub-scales as well as total CORE score able to be analyzed^51^.

### Natural stimuli – film clips

For the positive condition, participants viewed a stand-up comedy routine by Bill Cosby (taken from “Bill Cosby, Himself”). For the negative condition, participants viewed a scene from the drama film “The Power of One”, which depicts the inhumane treatment of prisoners under the apartheid era. For the neutral condition, participants watched dynamic video footage of landscapes (recorded from a moving plane) and moving streams of water with matching audio streams. Participants watched the films through an MRI-compatible monitor, and were provided with brief instructions (displayed on the screen) prior to the onset of each movie (“Video will begin soon. Please relax and watch.”). High-quality audio from the movies was provided through insert earphones using an MRI-compatible system (Sensimetrics Model S14). All video stimuli are available from the authors on request.

### Image acquisition and preprocessing

Structural and functional MR images of healthy controls and melancholic-MDD patients were acquired on a Philips 3 Tesla scanner equipped with a 12-channel receiver head coil. Functional images were pre-processed using the SPM8 toolbox^52^ in MATLAB (The MathWorks, Inc) following standard pipelines^29^,^53^. These preprocessed images were then used for inter-subject correlation and independent component analyses, as described in the following sections. Melancholic-MDD and HC groups did not differ in head motion profiles (two-sample t-test, p > 0.1). See SI for detailed acquisition and preprocessing parameters.

## Additional Information

**How to cite this article**: Guo, C. C. *et al.* Out-of-sync: disrupted neural activity in emotional circuitry during film viewing in melancholic depression. *Sci. Rep.* **5**, 11605; doi: 10.1038/srep11605 (2015).

## Supplementary Material

Supplementary Information

## Figures and Tables

**Table 1:** Participant demographics.

	**Healthy Controls**		**Patients with MDD**		**HC - MDD**
	**Mean (SD)**	**n**	**Mean (SD)**	**n**	***P*** **values**
Age, years	40.0 (13.3)	23	42.1 (11.8)	17	0.66
Gender (M:F)	11:12		8:9		0.96
Smoker (Y:N)	3:20		3:14		0.69
QIDS, total	1.3 (1.5)	21	16.8 (4.2)	17	<0.001
GAF	9.0 (0.2)	21	5.3 (0.9)	15	<0.001
CORE, total	0.0 (0.0)	21	10.3 (6.9)	17	<0.001
Age at onset, yrs	—		17.4 (10.1)	17	—
Duration of current episode, weeks	—		180 (398)	17	—
Family history (Y:N)	—		14:3		—
Current Medication, % yes (n)					
Nil medication	—		11.7% (2)		
Selective serotonin reuptake inhibitor	—		17.6% (3)		
Dual-action antidepressant*	—		58.8% (10)		
Mood stabilizer#	—		17.6% (3)		
Antipsychotic	—		47.1% (8)		

^*^e.g., serotonin noradrenaline reuptake inhibitor.

^#^e.g., lithium or valproate/divalproex.
