# Supplementary material for: Out-of-sync: disrupted neural activity in emotional circuitry during film viewing in melancholic depression
Source: Sci Rep. 2015 Jun 26;5:11605. doi: 10.1038/srep11605 (PMC4481375; doi:10.1038/srep11605)
Supplement: Supplementary Information [file srep11605-s1.doc]

Out-of-sync: disrupted neural activity in emotional circuitry during film viewing in melancholic depression

Christine C. Guo^1^, Vinh T. Nguyen^1^, Matthew P. Hyett^1,2^, Gordon B. Parker^2,3^ and Michael J. Breakspear^1,3^

1 QIMR Berghofer Medical Research Institute, Herston, Queensland, Australia

2 School of Psychiatry, University of New South Wales, Sydney, Australia

3 Black Dog Institute, Sydney, Australia

Address correspondence to:

Christine C. Guo, PhD

christine.cong@gmail.com

QIMR Berghofer

Locked Bag 2000

Royal Brisbane Hospital

Herston, Qld 4029

Australia

**Supplementary Figure 1.**


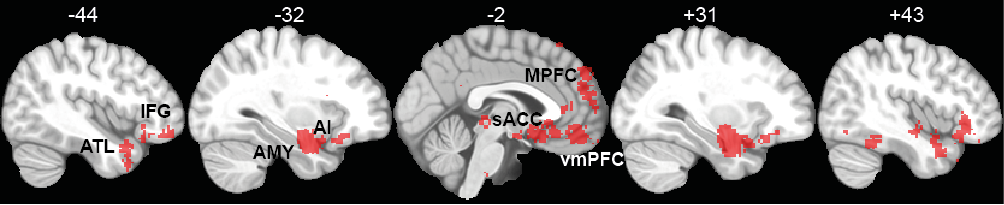


sFigure 1. Brain regions that are preferentially active in neuroimaging studies that load highly on the term "emotional" (FDR < 0.01). Results are generated by a meta-analysis on 1224 neuroimaging studies that contained the term ‘emotional’, implemented by the online platform – <http://neurosynth.org/> ^1^. AI = anterior insula; AMY = amygdala; ATL = anterior temporal lobe; IFG = inferior frontal gyrus; MPFC = medial prefrontal cortex; sACC = subgenual anterior cingulate cortex; vmPFC = ventromedial prefrontal cortex.

**Supplementary Figure 2.**


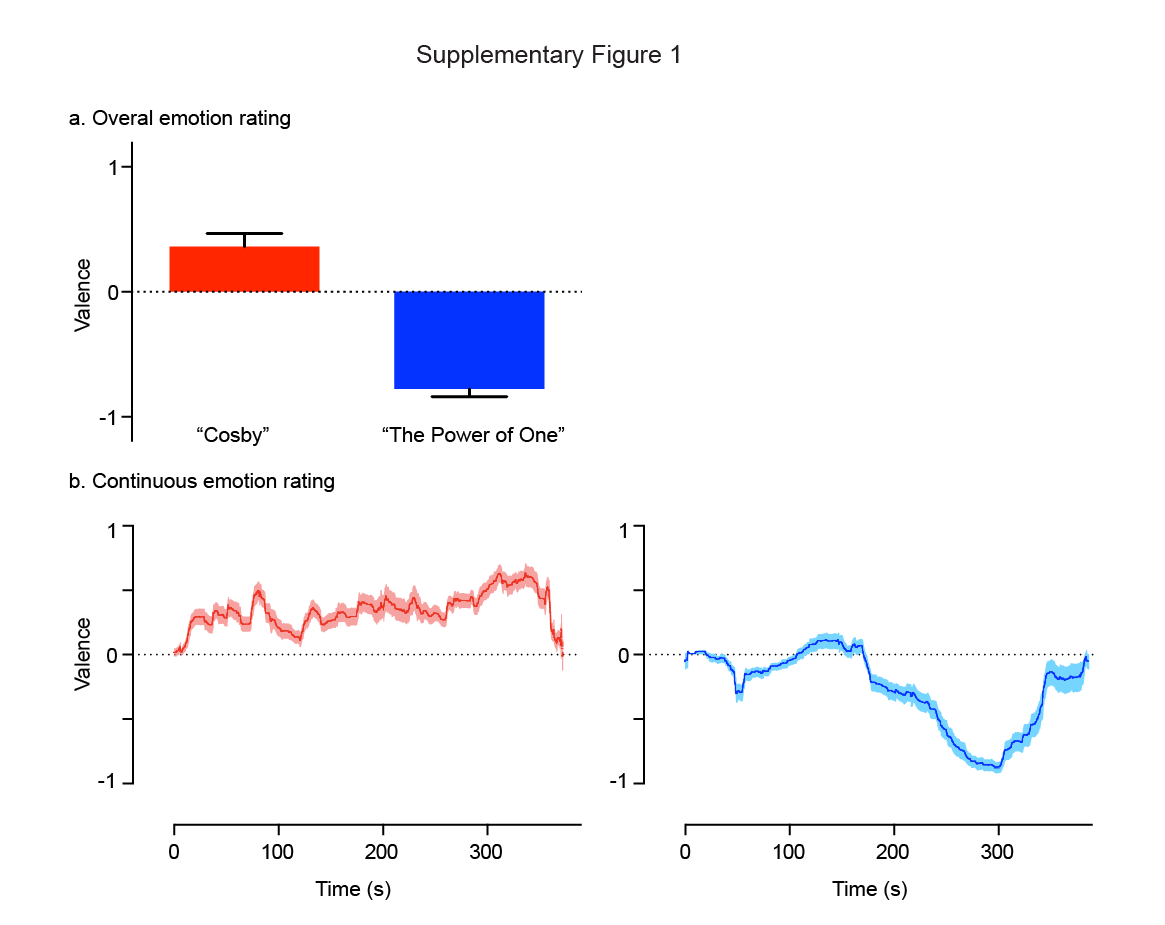


**sFigure 2.** a) Overall and b) continuous ratings of emotional valence for the two film clips, “Bill Cosby” and “The Power of One”, averaged across 18 healthy participants. Error bars signify standard error mean (SEM).

**Supplementary Figure 3.**

**
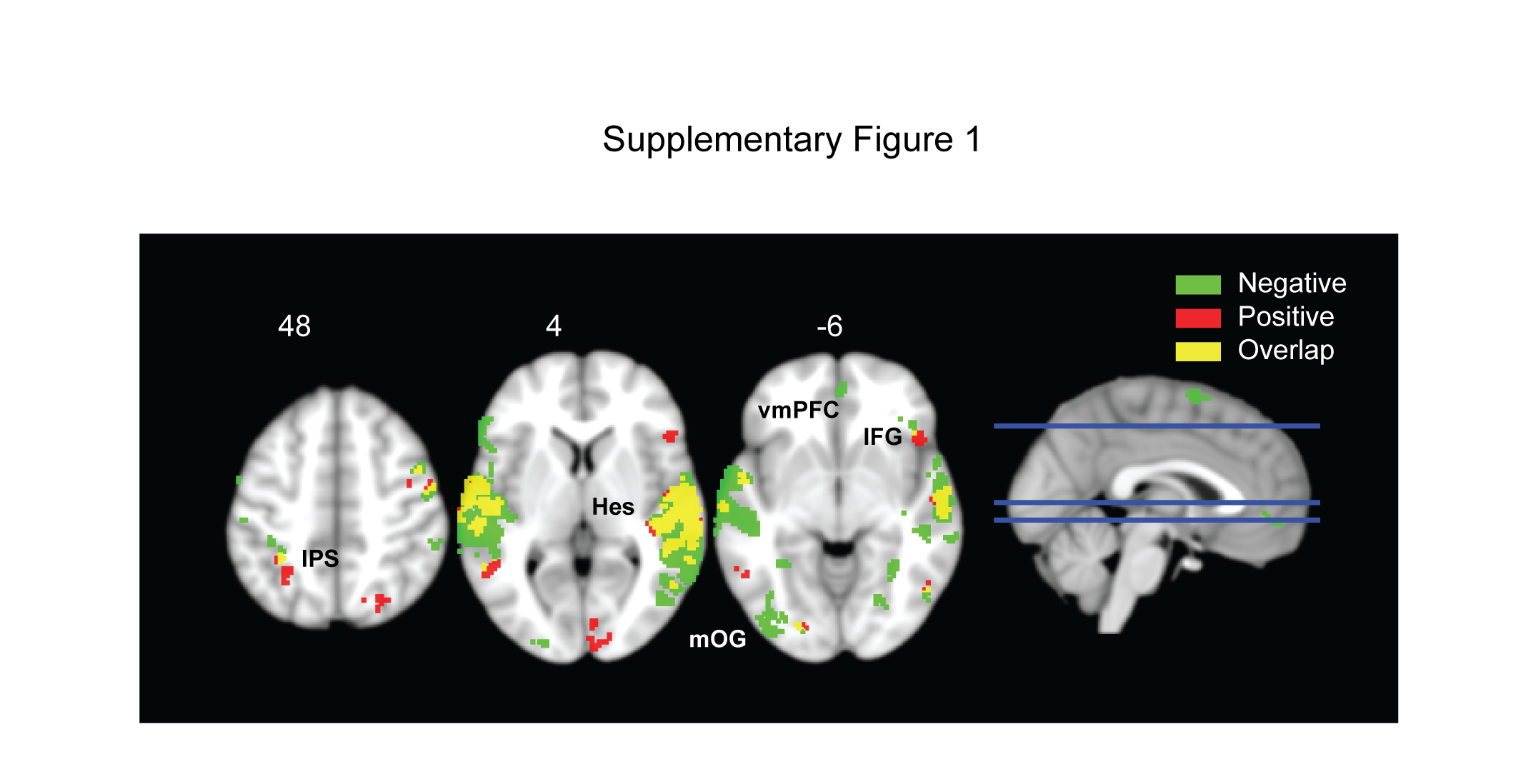
**

**sFigure 3.** Interaction effects (group x film) of ISC results. Statistical results on the interaction between group (HC and MDD) and film (negative and neutral, green; positive and neutral; red), and the overlap between the two tests (yellow; p < 0.05, FDR corrected; cluster size > 50 voxels). IFG = inferior frontal gyrus; IPS = intraparietal sulcus; Hes = Heschl’s; mOG = middle occipital gyrus; vmPFC = ventromedial prefrontal cortex.

**Supplementary Figure 4.**

**
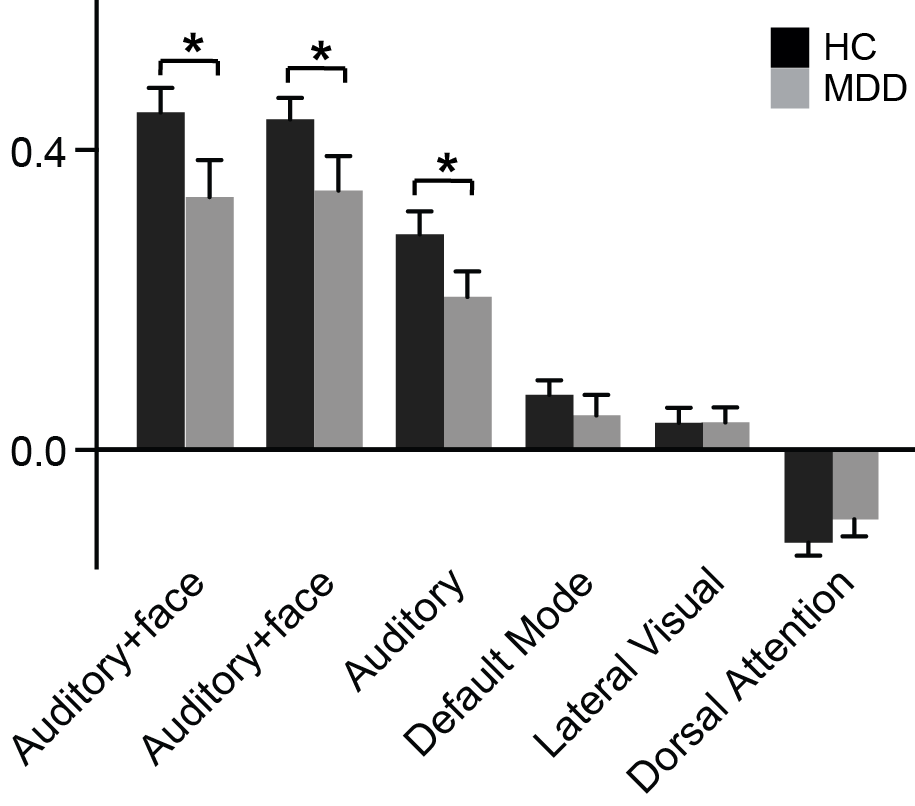
**

**sFigure 4.** Correlation coefficients between ICA component timeseries and estimated BOLD responses to face and voice, averaged separately for HC (black) and MDD (gray) groups. Error bars signify SEM. Asterisks signify significant differences between HC and MDD (p < 0.01).

**Supplementary Table 1.** Brain regions that are known to be involved in emotion processing. (Citations refer to the ones in the main text).

| Brain region | BA | Emotion processing functions |
| --- | --- | --- |
| Amygdala |  | Fear; salience detection ^37^ |
| Anterior Insula | 13 | Interoceptive awareness; disgust; fear. ^34,37^ |
| Medial Prefrontal Cortex | 9,10 | Emotion regulation^37^ |
| Subgenual ACC | 25 | Sadness^37^ |
| VmPFC | 10,32 | Visceral responses to emotional stimuli; reward-guided behaviours^35^ |

**Methods and Material**

**Formal diagnostic criteria for melancholia**. For a diagnosis of melancholia, two compulsory criteria were required: (A) psychomotor disturbance (expressed as motor slowing and/or agitation); and (B) an anhedonic mood state. In addition, five of the following nine clinical features were required (and met) in all assigned melancholic patients: (1) concentration and/or decision making impairment; (2) non-reactive affect; (3) distinct anergia; (4) diurnal mood variation – being worse in the morning; (5) appetite and/or weight loss; (6) early morning wakening; (7) no preceding stressors accounting for the depth of the depressive episode; (8) previous good response to adequate antidepressant therapy; and (9) normal personality functioning. Whilst respecting the DSM diagnostic approach to melancholia, these have been customised by our group to take into account criteria that has historically characterised melancholia ^2,3^.

**Emotion rating.**18 independent healthy participants provided emotion ratings for the Bill Cosby and “The Power of One” films. While viewing the film, participants provided continuous ratings of their emotion using rating software custom-built in LabView. They were instructed to continuously report their emotion while moving a mouse while they viewed the film. They were instructed to move the mouse cursor all the way to the left if they felt completely sad, depressed, disgusted or unpleasant; and move the mouse all the way to the right if they felt completely happy, joyful and pleased. A vertical bar, indicating their current rating (between -1 and 1), provided visual feedback. Negative ratings corresponded to values towards -1, whilst positive ratings corresponded to values towards +1. In addition, participants provided an overall rating of the film immediately after the viewing. The order of film presentation was counter-balanced between participants.

**Image acquisition**. A volumetric magnetization prepared rapid gradient echo (MP-RAGE) sequence was used to obtain T1-weighted images of the entire brain (repetition time/echo time/inversion time = 5.4/2.4/1000 ms, a bandwidth of 333.5 Hz/pixel, flip angle of 8°, capital orientation with a FOV = 256 x 256 mm; slice = 180; voxel size = 1 x 1 x 1 mm). Functional MRI scans were obtained using 33 transverse slices (3.2 mm thick) parallel to the plane connecting the anterior and posterior commissures and covering the whole brain using a T2*-weighted gradient echo–echo planar sequence (repetition time/echo time = 2000/30 ms; flip angle = 76°; FOV = 240 × 240 mm; matrix size = 120 × 120; voxel size = 2 × 2 × 3.2 mm; slice gap = 0 mm). All subjects underwent four sessions of fMRI scanning (each lasting 6 minutes and 15 sec). During three sessions (film-viewing fMRI sessions), the participants were instructed to lie still and freely watch the videos. During the resting state session, they were instructed to remain awake with their eyes closed. Resting-state data were acquired first from all subjects, while the remaining film-viewing fMRI sessions were pseudo-randomly presented and counterbalanced across participants.

**Data preprocessing**. The first eight images were discarded to allow for quasi-equilibrium in longitudinal magnetization to be achieved, while the films commenced with the ninth image. The remaining functional images were realigned and unwarped, slice-time corrected, co-registered, spatially normalized to standard space and smoothed with a 4 mm full-width at half-maximum Gaussian kernel. Co-registration was performed between the mean EPI images and the subject’s own T1-weighted image. Normalization and smoothing were carried out in one step using the DARTEL toolbox. Subsequently, the functional images were re-sampled at a voxel size of 2 mm^3^. These preprocessed images were then used for inter-subject correlation and independent component analyses, as described in the following sections.

**Head motion assessment***:* The motion parameters estimated in the process of realignment were used to compute the magnitude of head motion during each scan. Based on the volume-to-volume changes in motion parameters, mean root-mean-square (RMS) values were calculated for translation and mean Euler angles measured for rotation, as these summary metrics have been shown to correlate with network connectivity strength ^4^. Two-sample t-tests confirmed that the MDD and HC groups did not differ in translational (*p* = 0.94) or rotational movement (*p* = 0.87).

**Correlation between ICA timeseries and audio-visual streams.** To extract the relevant audio-visual features, we annotated the film “The Power of One” for the presence of both human faces and voices. The segments with both face and voice were time-stamped as “1” and the remaining segments as “0”. This time series was then convolved with the canonical hemodynamic response function to approximate BOLD responses to the conjunction of human face and voice. Pearson’s correlation coefficient was then used to assess the similarity between individual participant’s ICA time series and this convolved response. The average correlation coefficients from HC and MDD groups for the top five ICA components with the highest correlation are shown in sFig. 4.

**Tensor Independent Component Analysis (tensor-ICA).** Tensor Independent Component Analysis (tensor-ICA) provides a data-driven analysis of multi-subject and multi-session fMRI data. Tensor-ICA assumes that the temporal response pattern is the same across the population and provides a single decomposition for all original data sets. For each film stimulus, all subjects’ pre-processed images were entered into the MELODIC in FSL software ^5,6^. Principal components analysis (PCA) was used to reduce the data dimensionality for each subject. Each subject's reduced data were then concatenated to form a new 3-dimension dataset that was then decomposed into three matrices, denoting the factor loadings in the temporal, spatial and subject domain. This 3-dimension dataset was entered into a probabilistic ICA algorithm to calculate spatially independent components. The number of components was automatically estimated using the default parameters in MELODIC. For each film, tensor ICA identified 39, 33, and 38 components during natural viewing of the negative film, positive film and neutral film, respectively. Components were sorted in decreasing order of the median response per component, reflecting the extent to which each component is engaged among participants. Finally, the weighted mean time course of each component was calculated for each subject and entered into an inter-intra subject correlation analysis. The similarity between tensor ICA components and previously identified resting state networks was determined by spatial cross-correlation as implemented in fslcc ^7^ and confirmed by visual inspection.

**Inter-subject Correlation (ISC) analysis.** Voxel-wise ISC analysis was performed using the ISC toolbox ^8^. Briefly, for each condition, the Pearson correlation was calculated on the BOLD time series between each pair of participants within the HC or MDD groups on a voxel-by-voxel basis. For our ISC analysis on tensor ICA components, the Pearson correlation was calculated on the time courses of the ICA components derived from the tensor ICA analysis.

**Statistical analysis.** As correlation maps derived from each pair of participants are not independent, non-parametric permutation tests were used to identify statistical significance in results from ISC analyses, as implemented in the ISC toolbox ^8^. The null distribution was generated from 100 million permutation realizations. In each realization, the BOLD time course of each subject was circularly shifted by a random amount and ISC was recalculated on the shifted data. This practice preserves the temporal autocorrelation in the BOLD signals but disrupts the correlations between subjects. To identify voxels with significantly ISC values in healthy controls, we computed the p-values of each voxel based on the null distribution. These p-values were subsequently adjusted using false discovery rate (FDR) to correct for multiple comparisons. Significant voxel-wise ISC results were thresholded at p < 0.001 FDR corrected (Fig. 1a).

To compare differences in voxel-wise ISC maps between HC and MDD participants, we employed a non-parametric method implemented in the Statistical Non-Parametric Mapping toolbox ^9^. Two-sample permutation tests with 5000 permutations were used to test for group difference in ISC maps for negative, positive and neutral conditions, respectively (Fig. 1b). Two-group repeated measures factorial designs were used to test the interaction effect of group and video (negative vs. neural and positive vs. neutral, respectively; see Fig. 1). Maps with significant ISC differences were then generated using a threshold at p < 0.05 FDR corrected, with a spatial extent threshold of 50 voxels. Group differences in ICA component ISC results were tested using a two-sample permutation test with 5000 permutations. Statistical significance was FDR corrected for multiple comparisons at *p* < 0.05. **In addition, we calculated Bayes Factors as a measure of the robustness of the significant results revealed by the two-sample t-test (Rouder et al., 2009). This estimates the relative likelihood of the null versus the alternative hypothesis.**

Correlations between the variability of the vmPFC BOLD signal and the QIDS depression index were quantified by the Pearson’s coefficient. The average time course of BOLD fluctuations across voxels in the vmPFC clusters was extracted from each participant, and the variability was computed as the standard deviation of this time course. To examine whether the relationship between variability of BOLD signals and QIDS scores was due to non-specific noise, we generated a map that included all gray matter voxels outside of the ISC difference map (Fig. 1b). This map was used to extract the average BOLD signals of these non-specific brain regions for each participant, whose variability was then examined.

1. Yarkoni, T., Poldrack, R. a, Nichols, T. E., Van Essen, D. C. & Wager, T. D. Large-scale automated synthesis of human functional neuroimaging data. *Nat. Methods* (2011). doi:10.1038/nmeth.1635

2. Parker, G. *et al.* Issues for DSM-5: whither melancholia? The case for its classification as a distinct mood disorder. *Am. J. Psychiatry* **167,** 745–7 (2010).

3. Taylor, M. & Fink, M. *Melancholia: the diagnosis, pathophysiology and treatment of depressive illness*. (Cambridge University Press, UK, 2006). at <http://www.cambridge.org/us/academic/subjects/medicine/mental-health-psychiatry-and-clinical-psychology/melancholia-diagnosis-pathophysiology-and-treatment-depressive-illness?format=HB>

4. Van Dijk, K. R., Sabuncu, M. R. & Buckner, R. L. The Influence of Head Motion on Intrinsic Functional Connectivity MRI. *Neuroimage* 1–8 (2011). doi:10.1016/j.neuroimage.2011.07.044

5. Beckmann, C. F. & Smith, S. M. Probabilistic independent component analysis for functional magnetic resonance imaging. *IEEE Trans. Med. Imaging* **23,** 137–52 (2004).

6. Beckmann, C. F. & Smith, S. M. Tensorial extensions of independent component analysis for multisubject FMRI analysis. *Neuroimage* **25,** 294–311 (2005).

7. Smith, S. M. *et al.* Correspondence of the brain’s functional architecture during activation and rest. *Proc. Natl. Acad. Sci. U. S. A.* **106,** 13040–5 (2009).

8. Kauppi, J.-P., Jääskeläinen, I. P., Sams, M. & Tohka, J. Inter-subject correlation of brain hemodynamic responses during watching a movie: localization in space and frequency. *Front. Neuroinform.* **4,** 5 (2010).

9. Nichols, T. & Holmes, A. Nonparametric permutation tests for functional neuroimaging: a primer with examples. *Hum. Brain Mapp.* **25,** 1–25 (2002).
